# Rhizobial Chemotaxis and Motility Systems at Work in the Soil

**DOI:** 10.3389/fpls.2021.725338

**Published:** 2021-08-27

**Authors:** Samuel T. N. Aroney, Philip S. Poole, Carmen Sánchez-Cañizares

**Affiliations:** Department of Plant Sciences, University of Oxford, Oxford, United Kingdom

**Keywords:** rhizobia, *Rhizobium leguminosarum*, *Sinorhizobium meliloti*, *Azospirillum brasilense*, *Bradyrhizobium diazoefficiens*, motility, chemotaxis, soil

## Abstract

Bacteria navigate their way often as individual cells through their chemical and biological environment in aqueous medium or across solid surfaces. They swim when starved or in response to physical and chemical stimuli. Flagella-driven chemotaxis in bacteria has emerged as a paradigm for both signal transduction and cellular decision-making. By altering motility, bacteria swim toward nutrient-rich environments, movement modulated by their chemotaxis systems with the addition of pili for surface movement. The numbers and types of chemoreceptors reflect the bacterial niche and lifestyle, with those adapted to complex environments having diverse metabolic capabilities, encoding far more chemoreceptors in their genomes. The Alpha-proteobacteria typify the latter case, with soil bacteria such as rhizobia, endosymbionts of legume plants, where motility and chemotaxis are essential for competitive symbiosis initiation, among other processes. This review describes the current knowledge of motility and chemotaxis in six model soil bacteria: *Sinorhizobium meliloti, Agrobacterium fabacearum, Rhizobium leguminosarum, Azorhizobium caulinodans, Azospirillum brasilense*, and *Bradyrhizobium diazoefficiens*. Although motility and chemotaxis systems have a conserved core, rhizobia possess several modifications that optimize their movements in soil and root surface environments. The soil provides a unique challenge for microbial mobility, since water pathways through particles are not always continuous, especially in drier conditions. The effectiveness of symbiont inoculants in a field context relies on their mobility and dispersal through the soil, often assisted by water percolation or macroorganism movement or networks. Thus, this review summarizes the factors that make it essential to consider and test rhizobial motility and chemotaxis for any potential inoculant.

## Introduction

There are several strategies bacteria use to actively navigate their environment, motile forces produced either through pili retraction or flagella rotation. Pili drive twitching motility across surfaces by extending, adhering and retracting to pull the bacterium forward (Mattick, 2002), whereas flagella are rigid helical structures, anchored to the cell wall and rotated by a protein motor to produce thrust (Macnab and Aizawa, 1984). This enables the bacteria to move sporadically as individuals (swimming) or continuously as an organized group (swarming) (reviewed by Henrichsen, 1972). Alternatively, organized and continuous movement can be driven by motor proteins anchored to a surface or nearby bacterium, moving along helical tracks in the inner membrane and pushing the cell forwards (gliding) (Nan and Zusman, 2016). Other, passive strategies have also been described that are driven by the expansion of a growing culture, with the bacteria either producing substances to reduce friction and enable mass movement (sliding) or producing an aggregate capsule from which cells are ejected (darting) (Henrichsen, 1972; Pollitt and Diggle, 2017).

Active bacterial motility tends to be controlled by chemotaxis systems that respond to different stimuli, allowing bacteria to migrate to optimal environments. This occurs through sensing the binding of a ligand to its cognate chemoreceptors (methyl accepting chemotaxis proteins, MCPs). In response to signal transduction, motility systems produce runs, tumbles, reverses, pauses and other phenomena that together form a biased three-dimensional walk. Although tumbles, reverses and pauses are random reorientation events, the movement of bacteria is biased by controlling the frequency of these events. Some bacterial flagella rotate bidirectionally, others unidirectionally, to bias stopping and starting (Kearns, 2010).

An impressive diversity of motility mechanisms has evolved in prokaryotes. Among Gram-negative bacteria, *Escherichia coli* is the best understood model of flagella-based swimming. Also belonging to this group are a variety of nitrogen-fixing soil bacteria, known as rhizobia, that have evolved several differences from the *E. coli* model and are compared in this review. These include *Sinorhizobium meliloti* RU11 (also *Ensifer meliloti*), which nodulates alfalfa (*Medicago sativa*) and *Medicago truncatula* (Meade et al., 1982); *Agrobacterium fabacearum* H13-3 (formerly *R. lupini* H13-3), which nodulates *Lupinus luteus*, the European yellow lupin (Delamuta et al., 2020); *Rhizobium leguminosarum* bv. *viciae* 3841, which nodulates pea (*Pisum sativum*), various *Vicia*, lentils (*Lens culinaris*), grass peas and sweet peas (various *Lathyrus*) (Young et al., 2006); *Azorhizobium caulinodans* ORS571, which nodulates *Sesbania rostrata* (Lee et al., 2008); *Azospirillum brasilense* Sp7, a non-endosymbiote but a microaerobic diazotroph that colonizes the rhizospheres of grasses (Zhulin and Armitage, 1993); and finally, *Bradyrhizobium diazoefficiens* USDA110 (formerly *B. japonicum*), which nodulates soybean (*Glycine max*) (Kaneko et al., 2002). The relevance of motility and chemotaxis for rhizobia, both as free-living bacteria in the soil and as symbiotic cells inside plant nodules, is then discussed in the context of rhizobial inoculants and the importance of evaluating the chemotaxis and motility properties of strains used in the field.

### The *Escherichia coli* Motility Model and Marine Bacteria Motility

**Figure 1:**
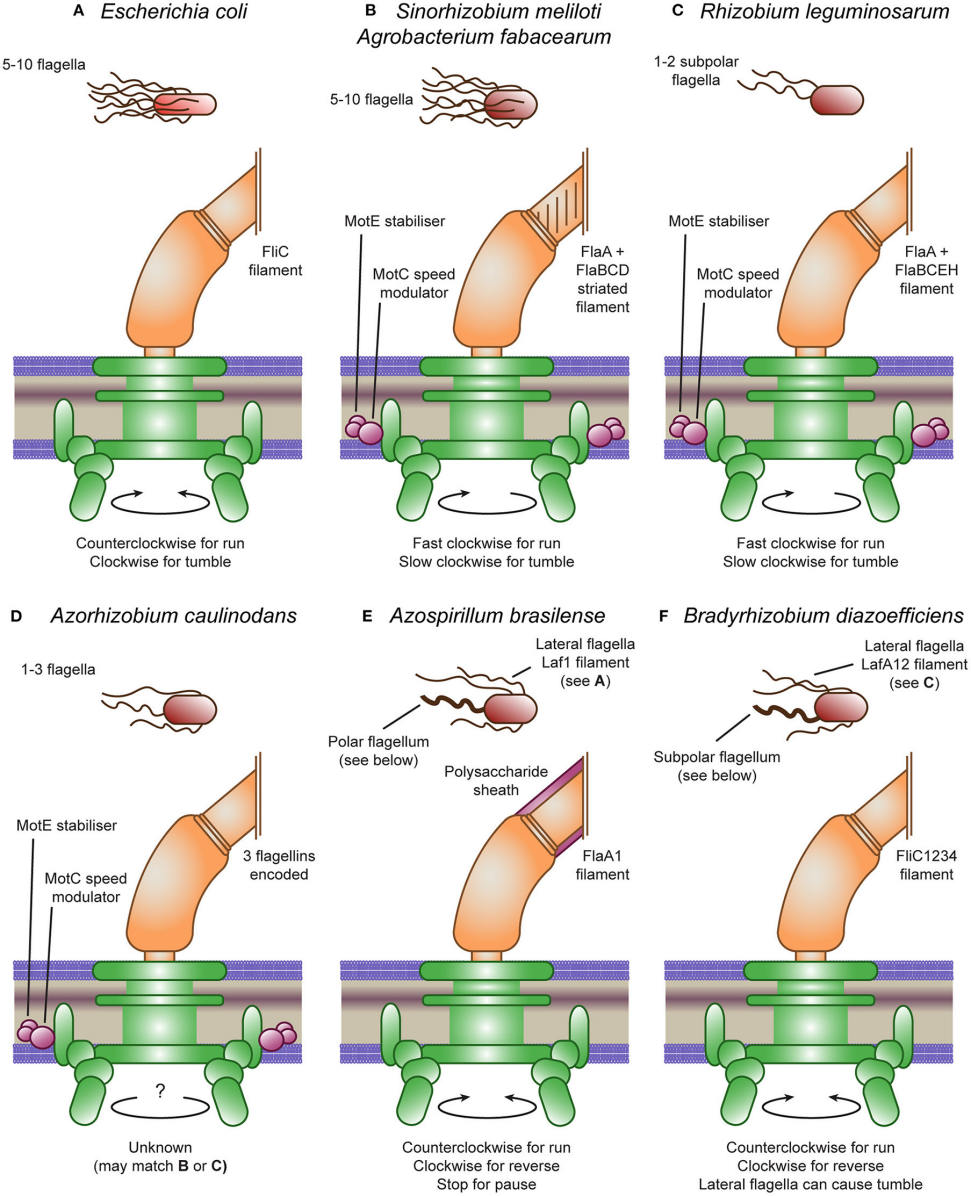
*Escherichia coli* and rhizobial motility models. **(A)** The *E. coli* peritrichous flagella provides thrust through counterclockwise rotation and tumbles through clockwise rotation causing the flagella bundle to dissociate. **(B)** The *Sinorhizobium meliloti* and *Agrobacterium fabacearum* peritrichous flagella provide thrust through clockwise rotation and tumbles through speed modulation causing the flagella bundle to dissociate. This speed modulation is controlled by MotC, stabilized by MotE. The reversed direction is due to the heterogeneous filament proteins producing a right-handed striated filament. **(C)** The *Rhizobium leguminosarum* subpolar flagella also provide thrust through clockwise rotation and tumbles through speed modulation, although they do not form a flagella bundle. The *R. leguminosarum* flagella also have heterogeneous filaments, although they are not visibly striated. **(D)** The *Azorhizobium caulinodans* flagella are not well-studied but may match either **(B)** or **(C)**, having the MotC and MotE accessory proteins. **(E)** *Azospirillum brasilense* produces two flagella types: a polar flagellum covered by a polysaccharide sheath that provides thrust through counterclockwise rotation, reverses through clockwise rotation and can pause by stopping the motor; and lateral flagella matching **(A)**. **(F)** *Bradyrhizobium diazoefficiens* produces two flagella types: a subpolar flagellum that provides thrust through counterclockwise rotation and reverses through clockwise rotation; and lateral flagella matching **(C)** that can cause tumbles.

*E. coli* typically have 5–10 flagella protruding from various points around the cell body (peritrichous flagellation) that form an integrated bundle oriented in the same direction (Macnab and Aizawa, 1984). Each flagellum consists of a basal body, hook and filament (see Figure 1A). The basal body is made up of a central hollow rod surrounded by anchoring stacks of rings (Wang et al., 2012). At the base of the basal body is the motor, where torque is produced via transmembrane gradients of H^+^ ions, and in other bacterial species, also of Na^+^ or K^+^ ions (Hirota and Imae, 1983; Armitage and Schmitt, 1997; Kojima and Blair, 2001; Terahara et al., 2012). This motor is further subdivided into the rotor, the proteins that rotate with the flagella, and the stator, the proteins driving rotation. The hook is a highly curved helix; a flexible coupling that connects the central rod to the filament. The filament is a left-handed helix consisting of a single protein, flagellin. When rotated in a counterclockwise (CCW) manner, the flagella provide a powerful forward thrust. However, if the rotary direction is flipped to a clockwise (CW) direction, a polymorphic change is induced that causes a right-angled bend in the filament to propagate along the flagella. This compels the bundle of flagella to separate, causing a period of rotational movement due to the low impact of inertia at microbial scales. This process is called a tumble and allows bacteria to change their direction of movement (Macnab and Aizawa, 1984; Armitage and Schmitt, 1997; Berg, 2003; Stock et al., 2012; Wang et al., 2012). The “run and tumble” mechanism produces a movement called a “biased random walk,” with the rate of tumbles being random but biased by the chemotaxis system (Sourjik and Wingreen, 2012).

In contrast, marine bacteria such as *Vibrio alginolyticus* tend to reverse their swimming direction instead of tumbling, by stably rotating their polar or subpolar flagella in the opposite direction and dragging the cell body. This “run and reverse” mechanism can also produce a “biased random walk” by controlling the frequency of reversal movements, although it is reliant on Brownian motion to reorient the cell body (Mitchell et al., 1996). *Vibrio* spp. (including *V. alginolyticus*) were recently discovered to also ‘flick' the cell body after a reversal, caused by buckling of the hook adapter, and resulting in a tumble-like random reorientation of the cell (Xie et al., 2011; Son et al., 2013; Taktikos et al., 2013). Indeed, up to 60% of marine bacteria may actually be performing “run, reverse, and flick” motility (Son et al., 2013).

### The Complex Flagella of *Sinorhizobium meliloti* and *Agrobacterium fabacearum*

*S. meliloti* and *A. fabacearum* are both well-studied Gram-negative swimming bacteria. Although their flagella are built in a similar fashion to those in *E. coli* and in similar numbers, there are several important differences (see Figure 1B). The main difference is the shape of the filament; *S. meliloti* and *A. fabacearum*'s filaments are formed by right-handed helices and as such forward thrust is produced by CW rotation (Gotz et al., 1982; Armitage and Schmitt, 1997; Sourjik and Schmitt, 1998). They also have increased rigidity, likely due to the use of several distinct flagellin proteins. Half of the flagella mass consists of the FlaA protein which forms heterodimers with two or three other proteins: FlaB, FlaD and, in *S. meliloti*, FlaC (Scharf et al., 2001). The rigid *S. meliloti* and *A. fabacearum* flagella may be adapted for use in the relatively viscous medium of soil, compared to the native habitat of *E. coli*, the human gut (Armitage and Schmitt, 1997; Attmannspacher et al., 2005). In addition, they are unable to switch the rotary direction of the motor. Instead, they modulate the speed of rotation through an extra stator protein, MotC, that binds MotB in the periplasm and may, thus, regulate the speed of the motor. A further additional protein, MotE, stabilizes MotC and may target the protein to the flagellar motor (Eggenhofer et al., 2004). The product of another nearby gene was originally designated as the novel motor protein MotD; however, mutational analysis revealed its function to be that of FliK, the flagellar hook length regulator that controls the switch from secretion of hook-type substrates to filament-type substrates (Eggenhofer et al., 2006).

Despite being unable to cause a tumble in the same way as *E. coli* due to their unidirectional flagella, *S. meliloti* and *A. fabacearum* can still tumble. The bacteria achieve this by asynchronously modulating the rotational speed of their flagella, causing their bundle of flagella to disassociate, causing a tumble (Armitage and Schmitt, 1997; Attmannspacher et al., 2005). The ability to modulate the speed of their flagella also enables the bacteria to increase cell velocity in addition to reducing tumbling in response to high attractant concentrations (Meier et al., 2007).

### The Smooth Flagella of *Rhizobium leguminosarum* and *Azorhizobium caulinodans*

Motility in *R. leguminosarum* and *A. caulinodans* appears to be closely related to that in *S. meliloti*, containing all the accessory genes (see Figures 1C,D). *R. leguminosarum* appear to only have 1-2 subpolar flagella with a smooth surface, lacking the helical perturbations found in *S. meliloti* (Tambalo et al., 2010a). *A. caulinodans* also have 1–3 smooth flagella, although they are arranged around the cell in a peritrichous arrangement (Liu et al., 2018a). The protein structure of the *R. leguminosarum* filament is closely related to *S. meliloti*, again consisting of heterologous pairs of a main flagellin (FlaA) and multiple secondary flagellin (FlaB, FlaC, FlaE, and FlaH) (Scharf et al., 2001; Tambalo et al., 2010a). Indeed, this bacterium is also unable to change the direction of rotation, so directional changes are driven by modulating the rotational speed of a single or pair of flagella, although the mechanism is currently unknown (Miller et al., 2007). However, *R. leguminosarum* still appears to tumble approximately every 3 s under homogeneous conditions, while it swims at approximately 38 μm/s (Miller et al., 2007). The protein structure of the *A. caulinodans* filament is unknown, but it does encode three flagellin copies. In addition, although the rotation direction of the flagella of *A. caulinodans* is unknown, since it encodes both *motC* and *motE*, it likely also modulates the speed of rotation (Lee et al., 2008).

### The Composite Flagellar Systems of *Azospirillum brasilense* and *Bradyrhizobium diazoefficiens*

Both *A. brasilense* and *B. diazoefficiens* encode two flagella systems, one producing a single polar or subpolar flagellum and the other producing multiple lateral flagella (see Figures 1E,F (Zhulin and Armitage, 1993; Kaneko et al., 2002). The flagellins forming the lateral flagella are encoded by *laf1* in *A. brasilense* and, *lafA1* and *lafA2* in *B. diazoefficiens* and are all 300–400 amino acids long, similar in size to other rhizobia (Moens et al., 1995a,b; Kanbe et al., 2007). In contrast, the flagellins forming the polar flagella are encoded by *fla1* in *A. brasilense* and, *fliC1, fliC2, fliC3*, and *fliC4* in *B. diazoefficiens* and are all 600–800 amino acids long, producing thicker filaments with bidirectional rotation (Zhulin and Armitage, 1993; Quelas et al., 2016). In addition, the lateral flagella of *B. diazoefficiens* were found to be more closely related to the flagella of other rhizobia than the divergent polar flagellum (Garrido-Sanz et al., 2019). Indeed, the *B. diazoefficiens* lateral flagella can only rotate in a single direction and their cluster also encodes *motC* and *motE* (Kanbe et al., 2007). The rotation direction of the *A. brasilense* lateral flagella is unknown but the cluster does not encode *motC* and *motE*. A further difference is that the polar flagellum of *A. brasilense* is covered with a polysaccharide sheath not found in other rhizobia (Moens et al., 1995b; Burygin et al., 2007; Belyakov et al., 2012). Glycosylation of flagella has been linked to avoiding plant immunity in *Pseudomonas syringae* (Takeuchi et al., 2003; Taguchi et al., 2009; Iwashkiw et al., 2013). In contrast, other rhizobial flagellin are sufficiently divergent from pathogenic flagellins to avoid a plant response (Felix et al., 1999). Perhaps the polar flagellum of *A. brasilense* requires glycosylation to prevent plant detection, in which case, the subpolar flagellum of *B. diazoefficiens* may be similarly glycosylated. Two flagellin modification genes, *flmA* and *flmB*, were found to be essential for the formation of the *A. brasilense* polar flagellum (Rossi et al., 2016). In addition to having a similar composite system, the physical properties of the *A. brasilense* and *B. diazoefficiens* lateral flagella and separately their polar flagella were found to be nearly identical and were classified into the same groups (Fujii et al., 2008). In contrast, the *S. meliloti* flagella were divergent from these groups, potentially due to their complex, striated structure (Fujii et al., 2008).

The swarming motility of both *A. brasilense* and *B. diazoefficiens* are driven mainly by the lateral flagella, with *A. brasilense* only producing lateral flagella on solid or semi-solid surfaces (Zhulin and Armitage, 1993; Covelli et al., 2013). The swimming motility of *A. brasilense* is mixed, following mainly “run and reverse” and “run, reverse, and flick” motility with occasional pauses and reduced swimming speed in response to attractants (chemokinesis) driven mainly by the polar flagellum (Zhulin and Armitage, 1993; Mukherjee et al., 2019). The swimming motility of *B. diazoefficiens* is similarly mixed, following 50% “run and reverse,” 30% “run, reverse, and flick” and, interestingly, 20% “run and tumble” (Quelas et al., 2016). The reversals and flicks were driven by the polar flagellum, whereas the tumbles were produced by the lateral flagella. The composite flagellar systems of *B. diazoefficiens* thus produce a composite motility performance.

### Rhizobial Pili Systems

In addition to flagella, *S. meliloti, A. fabacearum, R. leguminosarum, A. brasilense* and *B. diazoefficiens* encode type IV pili on their chromosomes, with *S. meliloti* and *B. diazoefficiens* encoding additional, truncated clusters (Krehenbrink and Downie, 2008; Wibberg et al., 2011; Wisniewski-Dye et al., 2011; Zatakia et al., 2014; Mongiardini et al., 2016). The main cluster of *B. diazoefficiens* is split with the *tadE, tadF*, and *tadG* genes located almost 3 Mb away from the remaining genes (Mongiardini et al., 2016). *A. caulinodans* does not encode a pili system (Lee et al., 2008). Each species' main cluster displays high homology to the *tad* systems of *Aggregatibacter actinomycetemcomitans* and *Caulobacter crescentus* (Tomich et al., 2007; Clock et al., 2008; Imam et al., 2011). Beyond bioinformatic characterization, very little research has been conducted on rhizobial pili systems. *A. brasilense* was found to form polar pili bundles that are required for biofilm formation (Shelud'ko and Katsy, 2001; Wisniewski-Dye et al., 2011). Interestingly, deletion of *pilA1*, the integral pilin subunit of the chromosomal cluster of *S. meliloti*, was found to reduce competitive nodulation of *Medicago sativa* plants (Zatakia et al., 2014). In addition, the TadG protein of *B. diazoefficiens* has some sequence similarity to *Bradyrhizobium* lectin BJ38, which is important for soybean root adhesion (Loh et al., 1993; Ho et al., 1994; Mongiardini et al., 2016). This indicates that there is a role for pili in legume symbiosis, perhaps during root colonization.

### The *Escherichia coli* Chemotaxis Model

Chemotaxis involves regulating motility through variations in the concentration of metabolically-relevant chemicals, being either attractants (compounds which benefit the bacteria) or repellents (those compounds with negative effects). Bacterial size is insufficient to spatially sense concentration gradients at swimming speeds so, instead, concentrations are compared temporally (Porter et al., 2011). Bacteria bias their movements toward high concentrations of attractants by reducing their tumbles, and away either from low concentrations of attractants or high concentrations of repellents by increasing their tumbles.

**Figure 2:**
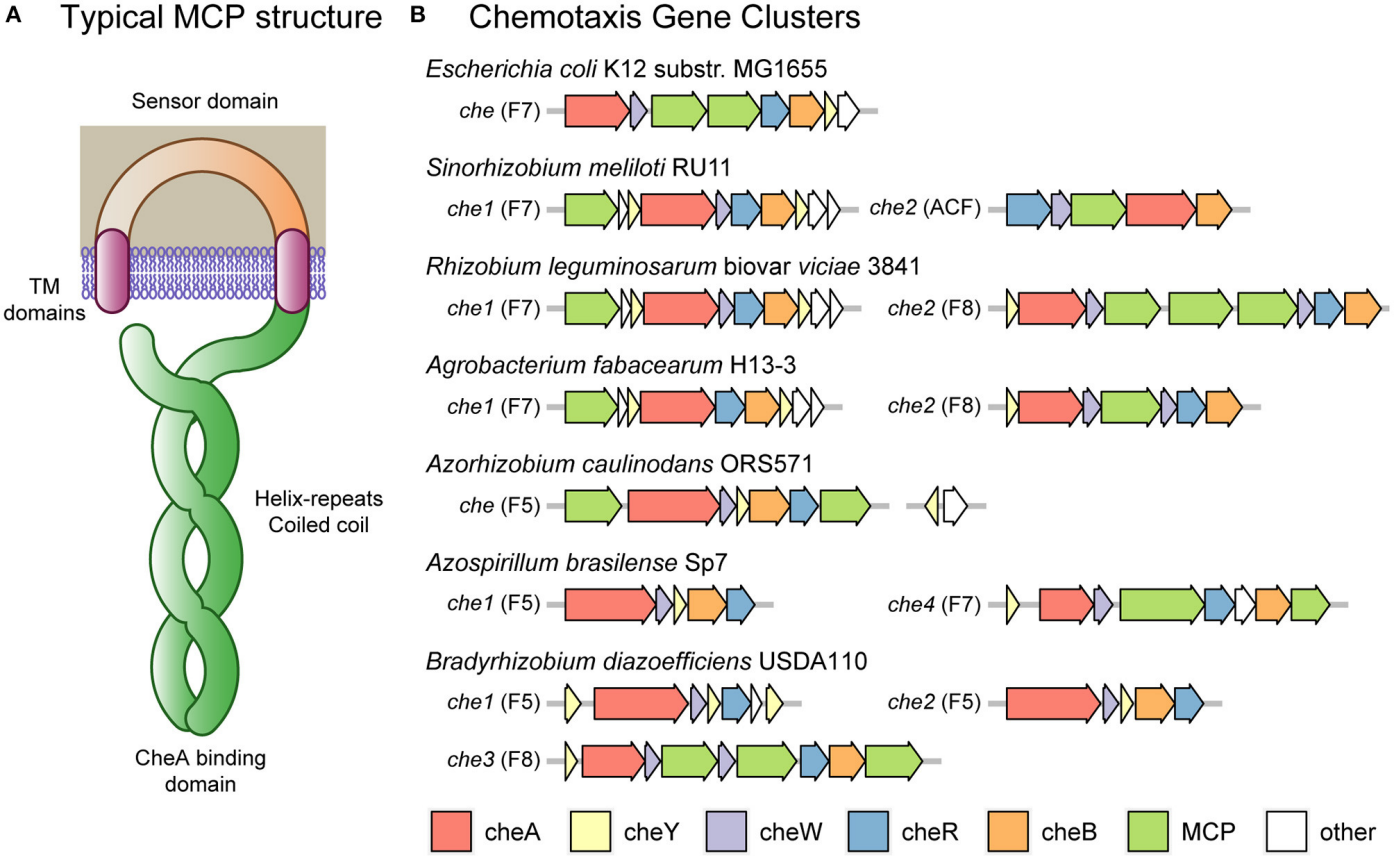
*Escherichia coli* and rhizobial chemotaxis systems. **(A)** Typical MCP (methyl-accepting chemotaxis protein) chemoreceptor domain structure including transmembrane domains flanking a periplasmic sensor domain. In the cytoplasm, the receptors form a coiled coil of alpha helices down to the CheA binding domain and returning to the plasma membrane to complete the coiled coil. Methylation and demethylation occur at sites along the coiled coil. The number of helix repeats varies, with some MCPs containing 34, 36, 38 or 40 repeats (classes 34H, 36H, 38H, and 40H, respectively). **(B)** The chemotaxis clusters of *Escherichia coli* and various rhizobia. Most clusters consist of the *che* genes *A, Y, W, R*, and *B*. *E. coli* K12 substr. MG1655 has one cluster of class F7 containing an extra *cheZ* gene and two MCP genes. *Sinorhizobium meliloti* RU11 has two clusters: one of class F7 containing extra *che* genes *Y, S, D*, and *T* and one MCP gene; and one of class ACF containing the standard genes except an altered *cheA*-REC, missing *cheY* and one MCP gene. *Rhizobium leguminosarum* biovar *viciae* 3841 has two clusters: one of class F7 containing extra *che* genes *Y, S, D*, and *T* and one MCP gene; and one of class F8 containing an extra *cheW* and three MCP genes. *Agrobacterium fabacearum* H13-3 has two clusters: one of class F7 containing extra *che* genes *Y, S, D*, and *T* and one MCP gene but missing *cheW*; and one of class F8 containing an extra *cheW* and one MCP gene. *Azorhizobium caulinodans* ORS571 has one cluster of class F5 containing the standard genes and one MCP gene; and 37 kb upstream the *che* genes *Y* and *Z*. *Azospirillum brasilense* Sp7 has four clusters: one of class F5 containing the standard genes; and one of class F7 containing the standard genes, *cheD* and two MCP genes. The two clusters not shown have not been linked to chemotaxis. *Bradyrhizobium diazoefficiens* USDA110 has three clusters: one of class F5 containing two extra *cheY* genes and one operon reading frame but missing *cheB*; one of class F5 containing the standard genes; and one of class F8 containing an extra *cheW* gene and three MCP genes.

The core of the chemotaxis response in *E. coli* is the phosphorylation of the response regulator CheY by the histidine kinase CheA in response to negative signal transduction from the chemoreceptor proteins. The chemoreceptors tend to sense in the periplasm and form a coiled coil of about 30–40 heptads (7 amino acid repeats) in the cytoplasm (forming the categories 34H, 36H, 38H, and 40H) and bind CheA at the base (Figure 2A) (Wuichet and Zhulin, 2010). The length of the coiled coil determines the association between chemoreceptors, with those of the same length forming hexagonal arrays (Jones and Armitage, 2015). The binding of CheA to the chemoreceptors within these hexagonal arrays is stabilized by CheW. Phosphorylated CheY binds the motor protein FliM, reducing rotary speed and inducing a tumble (Sourjik and Schmitt, 1998). In addition, the chemotaxis proteins CheR and phosphorylated CheB methylate and demethylate chemoreceptors, reducing and increasing their signal sensitivity, respectively (Rice and Dahlquist, 1991; Armitage and Schmitt, 1997; Porter et al., 2011). *E. coli* encodes an additional chemotaxis protein, CheZ, which interacts with CheY to remove phosphorylation, enabling the cell to quickly adapt to new conditions.

### Chemotaxis Systems in Symbiotic Bacteria

In contrast to *E. coli*, most of the rhizobia encode multiple chemotaxis systems. The rhizobia also encode a diversity of chemoreceptors divergent from the 5 chemoreceptors encoded by *E. coli* K12, with the motility model strain *S. meliloti* RU11 encoding 8 chemoreceptors, *A. fabacearum* H13-3 encoding 22, *R. leguminosarum* biovar *viciae* 3841 encoding 26, *A. caulinodans* ORS571 encoding 43, *A. brasilense* Sp7 encoding 41, and *B. diazoefficiens* USDA110 encoding 36 (Rebbapragada et al., 1997; Yost et al., 2004; Jiang et al., 2016; Scharf et al., 2016; Zatakia et al., 2017). However, the main chemotactic systems of *S. meliloti, A. fabacearum*, and *R. leguminosarum* have a similar core system to that of *E. coli*, belonging to class F7 and being associated with chemoreceptors of type 36H (Sourjik and Schmitt, 1998; Miller et al., 2007; Tambalo et al., 2010a,b; Wibberg et al., 2011) (see Figures 2A,B). These species do not encode CheZ, instead they encode two copies of CheY, with CheY2 propagating the signal and CheY1 acting as a phosphate sink, increasing the rate at which CheY2 returns to its unphosphorylated state (Sourjik and Schmitt, 1998). A further rhizobial protein, CheS, forms a dimer that binds CheA and CheY1, increasing phosphate transfer and thus CheY2 dephosphorylation (Dogra et al., 2012; Arapov et al., 2020). The rhizobia also encode two uncharacterized chemotaxis proteins: CheD and CheT. In the case of CheD, this protein has homology to *Bacillus subtilis* CheD protein which was found to deamidate chemoreceptors, a role served by CheB in *E. coli* (Kristich and Ordal, 2002; Rao et al., 2008). In *S. meliloti, cheT* mutants have a chemotaxis defect, indicating that it is likely to have some relevant, although currently unknown, chemotactic role (Arapov et al., 2020).

*A. caulinodans*, in contrast, only encodes a single chemotaxis system belonging to class F5 and being associated with chemoreceptors of type 38H. This bacterium encodes a cluster with *cheY, cheA, cheR, cheB, cheW* and two chemoreceptors (Jiang et al., 2016). In addition, it has a separate cluster located 37 kb upstream and encoding a second copy of the *cheY* gene and a *cheZ* gene. CheZ appears to be a functional protein with a conserved phosphatase motif critical for chemotactic activity (Liu et al., 2018b). Both of the CheY proteins appear to be active, with the main operon *cheY1* mutant displaying a reduced tumble rate, causing a 40% reduction in the chemotaxis swimming halo on semi-solid media, and a *cheY2* mutant displaying an increased tumble rate with a 90% reduction in the chemotaxis swimming halo (Liu et al., 2020).

In addition to the main systems described above, both *A. fabacearum* and *R. leguminosarum* encode accessory chemotaxis systems. These systems are F8-class chromosomal clusters encoding *cheY, cheA, cheR, cheB*, two *cheW* genes with one chemoreceptor (*mcpB*) in *A. fabacearum* and three in *R. leguminosarum*. These three chemoreceptors of *R. leguminosarum* (*mcrA, mcrB*, and *mcrC*) are of type 34H, indicating that it is an independent sensory system to the 36H chemoreceptors of Che1 (see Figure 2A). The class of the chemoreceptor encoded by *A. fabacearum* has not been determined, although this protein is likely to match *mcrA, mcrB*, and *mcrC* of *R. leguminosarum* due to the chemotaxis cluster arrangements. The *R. leguminosarum* Che2 cluster has a minor role in chemotaxis in free-living conditions (Miller et al., 2007). However, a recent insertion sequencing experiment in *R. leguminosarum* bv. *viciae* 3841 found that insertions in any of the *che2* cluster genes were over-represented in nodule bacteria samples, suggesting a role in symbiosis (Wheatley et al., 2020).

*S. meliloti* also encodes an accessory chemotaxis system located on its pSymA plasmid, which belongs to the alternative cellular function (ACF) class and encodes only *cheR, cheB, cheW*, a chemoreceptor (*mcpS*) and a *cheA* gene fused with a response-regulator (REC) domain (Wuichet and Zhulin, 2010; Scharf et al., 2016). This cluster is associated with a chemoreceptor of type 40H, *mcpS*, which is divergent from the other *S. meliloti* chemoreceptors of type 36H (see Figure 2A). The *mcpS* gene was not expressed in free-living cells, indicating that this chemotaxis operon is not active under those conditions (Meier and Scharf, 2009; Scharf et al., 2016). In addition, the absence of a *cheY* gene indicates that the system does not control flagella; instead, it is likely that the modified CheA-REC protein regulates downstream effectors.

### Chemotaxis Systems Controlling “Run and Reverse” Motility

In addition to multiple flagellar systems, *A. brasilense* has four chemotaxis clusters and *B. diazoefficiens* has three (see Figure 2B). Three of the four chemotaxis systems of *A. brasilense* (*che1, che2*, and *che4*) include the core *cheY, cheA, cheR, cheB*, and *cheW* genes. The *che1* system of *A. brasilense* is an F5-class cluster associated with chemoreceptors of type 38H (of which there are 33 in the genome), harboring the core genes above, including a *cheA* gene fused with a response-regulator (REC) domain (Wuichet and Zhulin, 2010). The *che2* system is an F9-class cluster containing the core genes mentioned above, including a fragmented *cheA* in addition to another *cheY* and a *cheC* phosphatase gene. The *che3* system is an ACF-class cluster only encoding a fragmented *cheA, cheB, cheW* genes, one chemoreceptor and a putative histidine-kinase response-regulator pair. Finally, the *che4* system is an F7-class cluster associated with chemoreceptors of type 36H (of which there are 8 in the genome), encoding the core genes and a *cheD* gene. *A. brasilense* has been observed to react with reverses and pauses and to modulate speed in response to chemical gradients (Zhulin and Armitage, 1993; Mukherjee et al., 2019). Numerous studies have revealed that the Che4 system modulates reversals by changing the rotation direction of the polar flagellum, while the Che1 system modulates swimming speed (Zhulin and Armitage, 1993; Bible et al., 2012; Mukherjee et al., 2016; Ganusova et al., 2021). In addition, the Che4 system, in concert with two orphan CheY proteins (CheY6 and CheY7), also modulates the pausing behavior, aerotaxis and the induction of swarming motility (Mukherjee et al., 2019; Ganusova et al., 2021). The Che2 and Che3 systems do not appear to be involved in chemotaxis. Regarding the three *B. diazoefficiens* chemotaxis clusters (*che1, che2*, and *che3*), they include the core *cheY, cheA, cheR, cheB*, and *cheW* genes, except the *che1* F5-class system, which does not contain a *cheB* gene but an additional two *cheY* genes. The *che2* system is also an F5-class cluster including the core genes. The *che3* system is an F8-class cluster associated with chemoreceptors of type 34H and encodes an additional *cheW* gene with three chemoreceptors (Kaneko et al., 2002; Wuichet and Zhulin, 2010). The involvement of these systems on chemotaxis is currently unknown, although it was found that only the subpolar flagellum of *B. diazoefficiens* responds chemotactically to glutamate and succinate (Quelas et al., 2016). This indicates that the primary chemotactic response is via the subpolar flagellum, similar to *A. brasilense*.

### Regulation of Motility and Chemotaxis

*S. meliloti, A. fabacearum*, and *R. leguminosarum* all have the same transcriptional activation system for flagellar and chemotaxis genes. Regulation of flagella in bacteria typically follows a hierarchical class system: regulatory class I genes control the transcription of class II genes required to initiate flagellar assembly and which, in turn, regulate class III genes to complete assembly and perform chemotaxis (McCarter, 2006). This class system ensures that each gene is active only when its product is useful. In the above rhizobia, the class I heterodimer VisNR is induced by some unknown effector to activate both promoters of *rem* and negatively regulate *visN* (Rotter et al., 2006). In turn, Rem activates the transcription of class IIA downstream regulators *fliM* (encoding a motor protein) and *orf38* (encoding a potential basal body protein) in addition to flagellar assembly and motor genes (class IIB) (Tambalo et al., 2010b). Presumably activated through some signal of basal body completion, FliM and Orf38 then activate flagellin and chemotaxis genes (class III), to produce functional flagella with chemotactic ability. Mutants in *visN, visR*, and *rem* are nonmotile and non-flagellated, indicating that they form the master regulators for flagella in *S. meliloti* and *R. leguminosarum* (Sourjik et al., 2000; Rotter et al., 2006; Tambalo et al., 2010b). Although all of these genes are encoded by *A. fabacearum*, their function has not yet been confirmed. In *R. leguminosarum*, VisNR and Rem are expressed throughout exponential growth phase, but their expression drops sharply during stationary phase; although the cells are still motile, likely retaining their flagella due to the stability of the filament (McCarter, 2006; Tambalo et al., 2010b; Zhuang and Lo, 2020; Nedeljkovia et al., 2021). The only motility and chemotaxis genes in *R. leguminosarum* that are independent of VisNR are scattered chemoreceptors and, notably, the *che2* cluster (Scharf et al., 2016). This possibly allows the bacteria to target the expression of *che2* genes independently of that of motility genes and indicates that the role of the Che2 cluster may not be linked to flagella.

Neither *A. caulinodans* nor *B. diazoefficiens* encode *visNR*, although both encode homologs of *rem* (Lee et al., 2008; Mongiardini et al., 2017). The *B. diazoefficiens* Rem-homolog LafR was recently found to control transcriptional regulation of its lateral flagella but not its polar flagellum. The orphan response regulator did not require phosphorylation for its effect although it was activated by arabinose and oxidative stress while being repressed by mannitol, oxygen limitation, and iron deficiency (Mongiardini et al., 2017). LafR was found to activate genes across multiple operons, including *flbT*, the gene product of which translationally induces the LafA1 and LafA2 lateral flagellins (Mongiardini et al., 2017). Although the role of *rem* in *A. caulinodans* is unknown, some recent work has indicated that the flagella and chemotaxis genes have increased transcription in the presence of the amino acids histidine, arginine and aspartate (Liu et al., 2019). *A. caulinodans* displays chemotaxis toward these amino acids, that are sensed by the chemoreceptor TlpH, although the *tlpH* mutant did not remove the transcriptional effect (Liu et al., 2019). The mechanistic link between the amino acids and flagellar transcriptional regulation remains unknown. In contrast to the other rhizobia, *A. brasilense* does not encode *visNR* or *rem*. Instead, it appears that the nitrogen regulator NtrA controls flagellar gene transcription via a σ^54^ box. Indeed, a *ntrA* mutant was found to be non-flagellated (Moens et al., 1995a).

### Importance of Chemotaxis and Motility Systems for Rhizobia as Free-Living Bacteria in Soil Conditions

**Figure 3:**
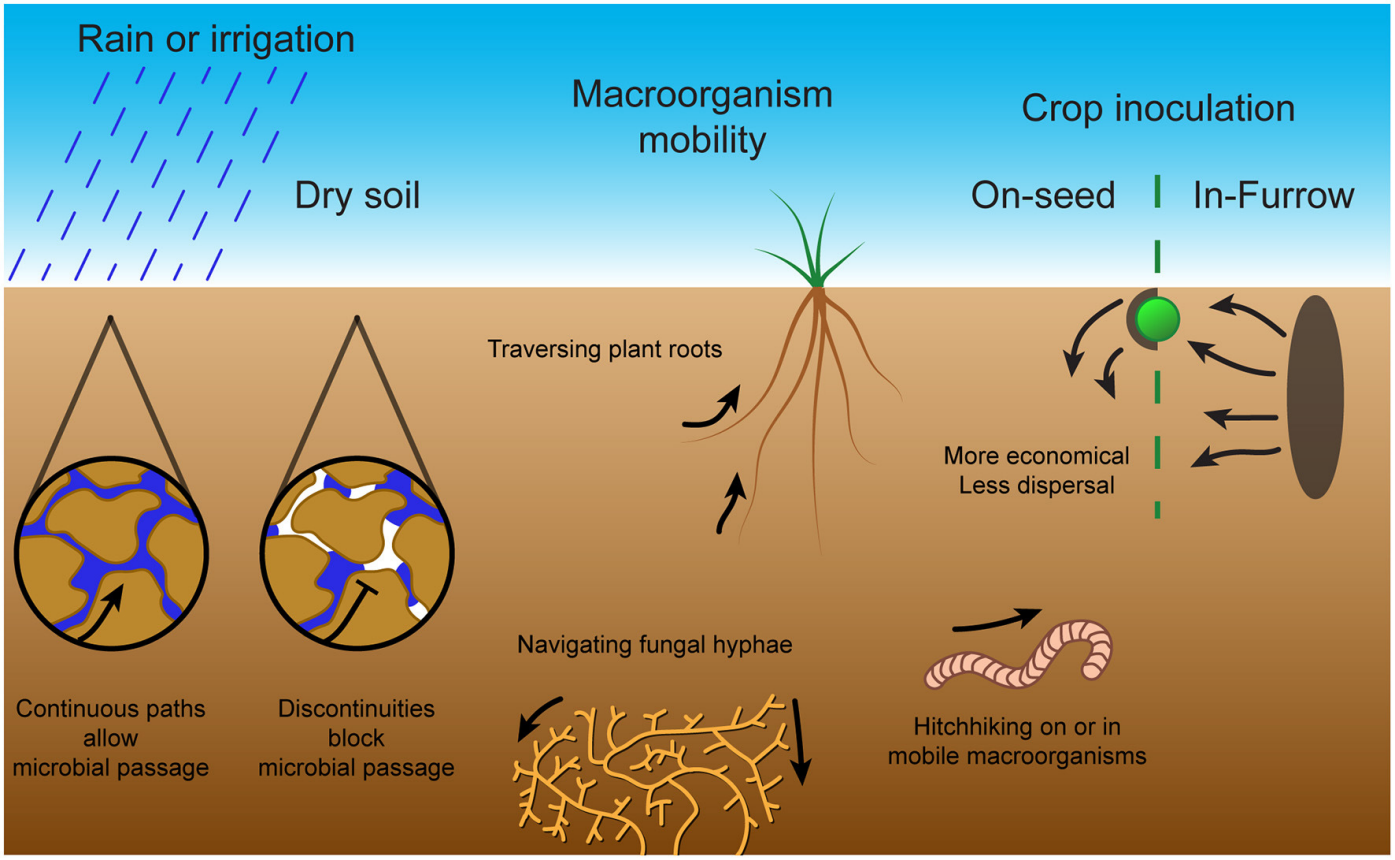
Bacterial mobility methods and challenges in soil. Microbial passage through the soil is dependent on sufficient moisture content to produce continuous pathways through water. Without rain or irrigation, air pockets form within the soil and prevent microbial passage. To combat this, microbes can exploit macroorganisms through methods including traversal along plant roots, navigation of fungal hyphae or hitching a ride on or in mobile macroorganisms. These mobility methods and challenges in soil are important for symbiont inoculation of crop plants. The two main inoculation techniques are on-seed, which although economical does not provide much dispersal, and in-furrow, which requires large amounts of inoculated soil but provides extensive dispersal.

Bacterial movement through soil depends on continuous water pathways with their inability to traverse air pockets (Griffin and Quail, 1968) (see Figure 3). Overall, with insufficient soil water content, microbial mobility is limited, whereas overloaded soil has greatly reduced oxygen concentrations (Tecon and Or, 2017). The water content in the soil has been described by different measures including matric potential, moisture content and percentage field capacity which all increase with increasing water content. For example, the swarming motility of various *Pseudomonas* species was found to be restricted to high matric potential agar with very high moisture content, showing a fast transition to non-motility upon drying (Dechesne and Smets, 2012). The soil bacterium *Pseudomonas aeruginosa* displayed reduced motility with decreases in soil water content (increased matric suction), to the point of non-motility (Griffin and Quail, 1968). *Rhizobium trifolii* motility also slowed with increasing water tension and was halted by discontinuity; conversely, the mean radius of the bacterial motility area increased with increased water content (Hamdi, 1971). The bean symbiont *Rhizobium leguminosarum* bv. *phaseoli* displayed a reduction in travel distance in soil with reduced moisture content such that it resembled a non-flagellated, non-motile mutant strain (Issa et al., 1993). The soil bacterium *Pseudomonas fluorescens* had reduced horizontal and vertical migration with decreased water irrigation flow rate (Singh et al., 2002). In addition, soil bacteria *A. brasilense* and *P. fluorescens* displayed increased migration in soil with large continuous pores (sand) over soil with narrow water channels (clay or loamy soils) (Bashan, 1986; Singh et al., 2002). The reduction in microbial dispersal with reduced soil water content implies that the usual state of soil bacteria is sessility imposed by the ubiquitous unsaturated state of soils (Tecon and Or, 2017). Thus, motility is limited in importance to temporary flooded states of soil formed immediately after rainfall or irrigation.

An effective rhizobial inoculant must survive in the competitive environment of the soil (Atieno and Lesueur, 2018). Water content was again found to be a key determinant for survival in the soil. Soil with higher moisture content and soil types with high field capacity (total water capacity of the soil) were found to support larger populations of *B. japonicum* and *R. leguminosarum* (Mahler and Wollum, 1981). *P. fluorescens* also had increased survival in soil with increased moisture content (van Elsas et al., 1991). At microbial scale, however, the soil is dynamic and fragmented with changes in soil hydration and pore-spaces dramatically influencing their dispersion and fluctuating nutrient gradients attracting them down fleeting paths. This produces a heterogeneity in microsites that is averaged out by macro studies at the cm^3^ scale (Tecon and Or, 2017). For instance, motile *E. coli* was found to accumulate within funnel-shaped microsites; thus, porous wet soil could cause heterogenous arrangements of bacteria simply by their shapes (Galajda et al., 2007). Theoretical models have indicated that in heterogeneous environments, a more motile bacteria can outgrow a metabolically superior species (Lauffenburger et al., 1982). Therefore, an elite inoculant would need to have powerful but adaptable motility and chemotaxis systems.

There are additional complications to chemotaxis control of motility in the soil. The control exerted by chemotaxis systems on bacterial motility does provide an improvement over random cell motility, with chemotactic strains having 10 times faster predicted soil dispersal rates (Ebrahimi and Or, 2014). Indeed, *P. fluorescens* was found to migrate further in soil irrigated by chemoattractant-rich fungal exudates when compared to sterile water (Singh et al., 2002). The soil bacteria *A. brasilense* and *P. fluorescens* migrate toward roots guided by chemotaxis at increased rates with increased water content up to the water capacity of the soil (Bashan, 1986). In contrast, the symbiont *B. diazoefficiens* was found to swarm quickly through wet soil with no supplemented chemoattractants (Covelli et al., 2013). Chemotactic detection of nutrients in soil is limited by fragmentation of water pockets due to drainage, plant uptake and evaporation, factors limiting nutrient diffusion and gradient establishment. Thus, reduction in soil matric potential—reducing moisture content—exponentially decreases chemotactic movement (Ebrahimi and Or, 2014).

Since active movement through dry soil is severely limited, other passive forms of movement dominate (see Figure 3). These include environmental forces, such as water percolation or wind dispersal of soil particles on the surface, in addition to “hitchhiking” on larger organisms such as earthworms, nematodes, protozoa, soil fungal networks or plant roots (Yang and van Elsas, 2018; King and Bell, 2021). This “hitchhiking” occurs through either adhesion to macroorganism surfaces, or through consumption and survival in the gut with subsequent release into the soil by expulsion or death of the macroorganism. For instance, *S. meliloti* migration through sterile soil was improved by the presence of *Caenorhabditis elegans* nematode worms, and migration of *B. japonicum* and *Pseudomonas putida* was improved by the presence of burrowing earthworms (Madsen and Alexander, 1982; Horiuchi et al., 2005). On the other hand, excessive adhesion may reduce dispersal, as *E. coli, Rhodococcus erythropolis* and *P. putida* species were found to be retained more in more adhesive soils with reduced vertical dispersal by water percolation (Jimenez-Sanchez et al., 2015; Sepehrnia et al., 2019). In addition, migration along hyphal networks or across plant roots enables mobility despite discontinuities in the surrounding soil pores (Yang and van Elsas, 2018). *Rhizobium leguminosarum* bv. *trifolii* was found to migrate further in presence of clover plant roots and *P. fluorescens* displayed greatly increased depth and magnitude of soil colonization in the presence of wheat plant roots (van Elsas et al., 1991; Worrall and Roughley, 1991). The range and depth of *A. brasilense* soil colonization was also increased by the presence of both wheat and weed roots (Bashan and Levanony, 1987). Thus, the importance of bacterial motility and chemotaxis in the soil is reliant on favorable environmental factors.

### Importance of Chemotaxis and Motility Systems for Plant Colonization and in Symbiosis

Motility and chemotaxis also appear to be crucial for competitive root surface colonization and for the establishment of a successful symbiosis (Catlow et al., 1990; Yost et al., 1998; Miller et al., 2007; Scharf et al., 2016). Indeed, most symbionts of eukaryotes have flagellar and chemotaxis capabilities (Raina et al., 2019). However, experiments were performed in well-watered pots that may not truly reflect the field environments in which these symbionts are applied as inoculants. There have been hints that macro-scale mechanisms, such as water percolation, are required to enable bacterial mobility and for effective colonization and nodulation. For example, in low-moisture soil, the symbiont *R. trifolii* inoculated on the seed displayed delayed infection thread formation and nodulation of *Trifolium subterraneum* plants that was partially recovered by watering (Worrall and Roughley, 1976). Vertical transportation of the symbiont *B. japonicum* and the soil bacterium *P. putida* in soil was found to be limited even in the presence of developing soybean and bean plant roots with the absence of percolating water or burrowing earthworms (Madsen and Alexander, 1982). The colonization and nodulation competitiveness of *R. leguminosarum* bv. *viciae* on lentil plants was also found to decrease with depth in non-sterile soil but to partially recover with increased soil moisture content (Karmakar and Chandra, 2012). Finally, various motile *B. diazoefficiens* strains (LP3001 and LP3008) out-competed their non-motile derivatives at soybean plant nodulation under flooded conditions but not when merely at field capacity (Althabegoiti et al., 2011).

A bacterium can achieve a growth advantage by arriving and establishing itself at the root surface before competing bacteria. In addition, flagella-based swarming motility enables movement along the surface of the root for more effective colonization (Simons et al., 1996; Gao et al., 2016). Indeed, motility and chemotaxis mutants of the soil bacterium *B. subtilis* display greatly reduced colonization on *Arabidopsis thaliana* roots after 4 h and on tomato roots after 2 weeks (Allard-massicotte et al., 2016; Gao et al., 2016). In addition, a *P. fluorescens* motile strain out-competed a non-motile strain in root colonization and attachment in sterile soil, whereas transfer of *A. brasilense* through sand from wheat to soybean roots was only observed with the motile strain (Bashan and Holguin, 1994; Turnbull et al., 2001). In contrast, *P. fluorescens* 2-79 RN10 was also found to traverse further along wheat plant roots when inoculated at the root apex rather than the seed, indicating that the bacteria moved with root growth (Parke et al., 1986). Thus, while motility and chemotaxis are integral systems for a competitively colonizing inoculant, they are not the sole mechanism for soil dispersal.

### Dispersal of Symbionts as Crop Inoculants in Field Conditions

Farming techniques alter soil microbial diversity and abundance by changing soil composition through crop cultivation or fertilizer application or by disrupting aggregate structures through tillage (Tecon and Or, 2017). Thus, it is important that any inoculants used are tested in real-world field use and are applied in an efficient and effective manner. Current inoculants tend to be applied either on-seed as a coating or in-furrow within the soil. Seed inoculation is more economical, so it has been used more extensively, although some studies indicate that in-furrow inoculation provides improved dispersal of the inoculant (Deaker et al., 2012; Iturralde et al., 2019). For instance, in field soil multiple symbiotic *B. japonicum* strains inoculated on-seed were found to have reduced nodule occupancy compared to in-furrow inoculation, particularly at depth and on lateral roots (McDermott and Graham, 1989; Wadisirisuk et al., 1989; Bogino et al., 2011). In addition, *B. japonicum* LP3001 established in vermiculite soil greatly out-competed (in soybean root nodulation) the equally competitive *B. japonicum* LP3004 inoculated on the seed (Lopez-Garcia et al., 2002). Interestingly, the seed-inoculated *Bradyrhizobium* had 50% reduced colonization in the mid root and apical root sections, such that slow vertical displacement of the seed-inoculated strain was identified as a major factor in the relative competitiveness of the strains (Lopez-Garcia et al., 2002). In further experiments with a hyper-motile strain, it was found that in-furrow inoculation of *B. japonicum* LP3008 could also considerably improve soybean root nodulation competitiveness compared to seed inoculation in field soil conditions (Lopez-Garcia et al., 2009). In addition, although on-seed inoculation of *R. leguminosarum* bv. *viciae* on faba bean and *Bradyrhizobium lupini* on lupin produced more and larger nodules than in-furrow inoculation at more than half of the field sites tested, potentially these nodules were larger because they were taproot nodules initiated earlier (Denton et al., 2017). Denton et al. (2017) also found that, in general, rhizobial inoculation improved taproot nodulation, whereas only improved lateral root nodulation in sites with reduced native rhizobia. Thus, the effectiveness of these two application techniques may depend on both the field conditions and the native rhizobial population. To improve the competitiveness of inoculants, other strategies include in-field dispersal in porous bags filled with inoculated soil which enable multiple establishment attempts or by leveraging interactions with motile organisms or fungal hyphal networks to promote dispersal (King and Bell, 2021). However, selecting for excess dispersal could actually be counterproductive by diluting inoculants away from the intended targets.

## Discussion

Regardless of the complexity and importance of motility and chemotaxis in the soil environment, there is a clear selection for chemotaxis genes in wildtype rhizobia. Analysis of 264 completed bacterial genomes gave an average chemoreceptor gene count of 14, which was found to be increased in species with high metabolic diversity, low stability of habitat and those with interactions with other living species (Lacal et al., 2010). The category with the highest number of chemoreceptors included soil-dwelling organisms such as *Azospirillum lipoferum* or *Bradyrhizobium* sp. strain BTAi1 encoding 63 and 60 predicted chemoreceptors, respectively, and several plant-interacting bacteria, including *A. caulinodans* ORS571 encoding 43 genes, *R. leguminosarum* bv. *viciae* 3841 encoding 26 genes and *B. diazoefficiens* USDA110 encoding 36 genes (Scharf et al., 2016). In contrast, *E. coli*, a human gut microbe, has only 5 chemoreceptor genes. A mutant of *Azorhizobium caulinodans* ORS571 with the chemotaxis cluster deleted was defective in *Sesbania rostrata* root surface colonization and competitive nodulation (Liu et al., 2018a). In *R. leguminosarum* biovar *viciae* VF39SM, deletion of the chemotaxis receptors *mcpB* or *mcpC* resulted in a significant reduction of nodulation competitiveness on pea plants, although the ability to nodulate was not affected (Yost et al., 1998). Importantly, the VF39SM *mcpC* mutant does not have a competitiveness disadvantage in symbiosis with other plants, indicating that plant specificity is based on the perception of the different exudates each plant secretes and which accordingly drive a defined chemotaxis response (Scharf et al., 2016). In the case of *S. meliloti* RU11, the chemoreceptors McpU and McpX have been found to target amino acids and quaternary ammonium compounds, respectively, being both present in the alfalfa host seed exudate (Webb et al., 2016, 2017a,b). Once inside the plant root, *S. meliloti* motility genes still appear to be expressed in bacteria in the infection thread channel but are completely down-regulated in bacteroids inside mature nodules (Yost, 1998). Interestingly, in other eukaryote-microbe symbioses, similar channels are generated to select for and guide their symbionts through generated gradients, via chemotaxis, toward the symbiosis space (e.g., the slime cavities of Hornwort or the ducts of the bobtail squid) (Raina et al., 2019). However, once inside the nodule, motility and chemotaxis are unnecessary for the now sessile bacteroids. This has been observed in *R. leguminosarum*, with down-regulation of chemoreceptors during bacteroid differentiation to undetectable amounts of expression in bacteroids (Yost et al., 2004; Karunakaran et al., 2009; Tambalo et al., 2010b). This regulation appears unrelated to the low oxygen conditions and organic acid availability inside the nodule (Yost et al., 2004; Tambalo et al., 2010b; Scharf et al., 2016). Thus, both motility and chemotaxis as dispersal strategies and competitive advantages are important aspects of symbiotic rhizobia.

As we have seen, chemotaxis and motility systems in rhizobia have a clear role both in free-living cells in soil and in symbiotic lifestyles, as indicated by their prevalence and the average number of receptor genes (Lacal et al., 2010; Scharf et al., 2016). However, bacterial movement through soil depends on continuous water pathways with their inability to traverse air pockets and, thus, the role of motility is limited to the temporary flooded states of soil formed immediately after rainfall or irrigation (Griffin and Quail, 1968). In addition, reduction in moisture content also decreases exponentially chemotactic movement by preventing the dispersal of chemoattractants and the formation of gradients (Ebrahimi and Or, 2014). Alternatively, environmental forces such as water percolation and the movement of larger organisms such as earthworms provide some degree of microbial dispersal, bypassing discontinuities in water channels (Yang and van Elsas, 2018; King and Bell, 2021). Another method used to bypass air pockets is traveling along eukaryotic pathways such as fungal hyphal networks and plant roots. Colonization of these networks can provide growth and settlement advantages, with chemotaxis and motility systems enabling the bacterium to arrive and establish before competing bacteria. Thus, although bacterial motility and chemotaxis are clearly important for soil competition, their importance is reliant on the favorable state of environmental factors.

The importance of motility and chemotaxis for elite inoculants—based on competitive and effective strains—is equally complex. Although motility and chemotaxis are integral systems for a competitively colonizing inoculant, their shortcomings mentioned above mean that they are not the sole mechanism for soil dispersal. It is thus important for any inoculant strain to be tested in field conditions and applied through efficient and effective methods. Of the two main techniques, it appears that on-seed inoculation is more economical but provides reduced dispersal opportunities, whereas in-furrow inoculation requires a larger quantity of the inoculant but provides superior dispersal along crop root systems (Deaker et al., 2012; Tecon and Or, 2017). The effectiveness of both application techniques also seems to depend on both the field conditions and the native rhizobial population. Therefore, both motility and chemotaxis should be considered factors in the selection of elite inoculants, together with other dispersal strategies.

## Conclusions

Improvements in symbiont inoculants depend on improvements in (1) understanding of motility and chemotaxis systems in rhizobia, (2) observations of motility and chemotaxis processes in the soil and (3) fast-throughput assays for individual inoculant selection. Firstly, more detailed knowledge of the chemotaxis and motility systems in rhizobia could determine the applicability of studies in other bacteria, such as *E. coli*, and enable mobility improvements to existing symbiont inoculants. For instance, improved knowledge of the many chemoreceptors with uncharacterized sensor domains could provide critical knowledge of the chemotactic signals that are critical for effective soil motility (Compton and Scharf, 2021). Additionally, assays that emulate field conditions, such as capillary and microfluidic assays, could be used to discover the decision-making logic of soil chemotaxis to allow targeted mimicry in engineered inoculants (Mao et al., 2003; Walsh et al., 2017; Lopez-Farfan et al., 2019). Secondly, improved observations of motility and chemotaxis processes in the soil would provide more detailed information about the heterogeneity of movement in the soil and rhizobial motility at the micro-scale. The micro-scale control of motility in the soil has not previously been directly observed. Potentially, transparent pseudo-soils could enable visual tracking of mobility and dispersal from different inoculation techniques toward crop plants (Bhattacharjee and Datta, 2019; Ma et al., 2019). Finally, inoculant selection could be improved by the development of fast-throughput assays that could assess the dispersal effectiveness of an inoculant. For example, the work by Mendoza-Suarez et al. (2020) describes a fast-throughput assay to test the competitiveness and effectiveness of strains in a particular soil. Similar experiments could be devised to measure the field dispersal properties of candidate strains for inoculant formulations. In addition, they could enable more precise testing of other inoculation strategies, such as porous bag embedding or interactions with motile macroorganisms (King and Bell, 2021). These improvements could enable a faster and more effective generation work-flow for selecting or engineering symbiont inoculants by clarifying the importance of effective soil dispersal and dynamic chemotaxis and motility systems.

## Author Contributions

SA, CS-C, and PP conceived the project and wrote and edited the manuscript. All authors contributed to the article and approved the submitted version.

## Conflict of Interest

The authors declare that the research was conducted in the absence of any commercial or financial relationships that could be construed as a potential conflict of interest.

## Publisher's Note

All claims expressed in this article are solely those of the authors and do not necessarily represent those of their affiliated organizations, or those of the publisher, the editors and the reviewers. Any product that may be evaluated in this article, or claim that may be made by its manufacturer, is not guaranteed or endorsed by the publisher.

## References

[B1] Allard-massicotte R. Tessier L. Lecuyer F. Lakshmanan V. Lucier J. (2016). *Bacillus subtilis* early colonization of *Arabidopsis thaliana* roots. mBio 7, e01664–16. 10.1128/mBio.01664-16 27899502 PMC5137498

[B2] Althabegoiti M. J. Covelli J. M. Pérez-Giménez J. Quelas J. I. Mongiardini E. J. López M. F. López-Garcia S. L. Lodeiro A. R. (2011). Analysis of the role of the two flagella of *Bradyrhizobium japonicum* in competition for nodulation of soybean. FEMS Microbiol. Lett. 319, 133–139. 10.1111/j.1574-6968.2011.02280.x 21470300

[B3] Arapov T. D. Saldaña R. C. Sebastian A. L. Ray W. K. Helm R. F. Scharf B. E. (2020). Cellular stoichiometry of chemotaxis proteins in *Sinorhizobium meliloti*. J. Bacteriol. 202:e00141–20. 10.1128/JB.00141-20 32393521 PMC7317046

[B4] Armitage J. P. Schmitt R. (1997). Bacterial chemotaxis: *Rhodobacter sphaeroide* and *Sinorhizobium meliloti*- variations on a theme? Microbiology 143, 3671–3682. 10.1099/00221287-143-12-3671 9421893

[B5] Atieno M. Lesueur D. (2018). Opportunities for improved legume inoculants: enhanced stress tolerance of rhizobia and benefits to agroecosystems. Symbiosis 77, 191–205. 10.1007/s13199-018-0585-9

[B6] Attmannspacher U. Scharf B. Schmitt R. (2005). Control of speed modulation (chemokinesis) in the unidirectional rotary motor of *Sinorhizobium meliloti*. Mol. Microbiol. 56, 708–718. 10.1111/j.1365-2958.2005.04565.x 15819626

[B7] Bashan Y. (1986). Migration of the rhizosphere bacteria *Azospirillum brasilense* and *Pseudomonas fluorescens* towards wheat roots in the soil. J. Gen. Microbiol. 132, 3407–3414.

[B8] Bashan Y. Holguin G. (1994). Root-to-root travel of the beneficial bacterium *Azospirillum brasilense*. Appl. Environ. Microbiol. 60, 2120–2131. 16349297 10.1128/aem.60.6.2120-2131.1994 PMC201610

[B9] Bashan Y. Levanony H. (1987). Horizontal and vertical movement of *Azospirillum brasilense* Cd in the soil and along the rhizosphere of wheat and weeds in controlled and field environments. Microbiology 133, 3473–3480. 10.1099/00221287-133-12-3473

[B10] Belyakov A. Y. Burygin G. L. Arbatsky N. P. Shashkov A. S. Selivanov N. Y. Matora L. Y.. (2012). Identification of an O-linked repetitive glycan chain of the polar flagellum flagellin of *Azospirillum brasilense* Sp7. Carbohydr. Res. 361, 127–132. 10.1016/j.carres.2012.08.019 23017779

[B11] Berg H. C. (2003). The rotary motor of bacterial flagella. Annu. Rev. Biochem. 72, 19–54. 10.1146/annurev.biochem.72.121801.161737 12500982

[B12] Bhattacharjee T. Datta S. S. (2019). Bacterial hopping and trapping in porous media. Nat. Commun. 10:2075. 10.1038/s41467-019-10115-1 31061418 PMC6502825

[B13] Bible A. Russell M. H. Alexandre G. (2012). The *Azospirillum brasilense* Che1 chemotaxis pathway controls swimming velocity, which affects transient cell-to-cell clumping. J. Bacteriol. 194, 3343–3355. 10.1128/JB.00310-12 22522896 PMC3434747

[B14] Bogino P. Nievas F. Banchio E. Giordano W. (2011). Increased competitiveness and efficiency of biological nitrogen fixation in peanut via in-furrow inoculation of rhizobia. Eur. J. Soil Biol. 47, 188–193. 10.1016/j.ejsobi.2011.01.005

[B15] Burygin G. L. Shirokov A. A. Shelud'ko A. V. Katsy E. I. Shchygolev S. Yu. Mato L. Yu. (2007). Detection of a sheath on *Azospirillum brasilense* polar flagellum. Microbiology 76, 728–734. 10.1134/S0026261707060124 18297874

[B16] Catlow H. Y. Glenn A. R. Dilworth M. J. (1990). Does rhizobial motility affect its ability to colonize along the legume root? Soil Biol. Biochem. 22, 573–575. 10.1016/0038-0717(90)90196-7

[B17] Clock S. A. Planet P. J. Perez B. A. Figurski D. H. (2008). Outer membrane components of the tad (tight adherence) secreton of *Aggregatibacter actinomycetemcomitans*. J. Bacteriol. 190, 980–990. 10.1128/JB.01347-07 18055598 PMC2223556

[B18] Compton K. K. Scharf B. E. (2021). Rhizobial chemoattractants, the taste and preferences of legume symbionts. Front. Plant Sci. 12:686465. 10.3389/fpls.2021.686465 34017351 PMC8129513

[B19] Covelli J. M. Althabegoiti M. J. López M. F. Lodeiro A. R. (2013). Swarming motility in *Bradyrhizobium japonicum*. Res. Microbiol. 164, 136–144. 10.1016/j.resmic.2012.10.014 23124116

[B20] Deaker R. Hartley E. Gemell G. (2012). Conditions affecting shelf-life of inoculated legume seed. Agriculture 2, 38–51. 10.3390/agriculture2010038

[B21] Dechesne A. Smets B. F. (2012). Pseudomonad swarming motility is restricted to a narrow range of high matric water potentials. Appl. Environ. Microbiol. 78, 2936–2940. 10.1128/AEM.06833-11 22327576 PMC3318784

[B22] Delamuta J. R. M. Scherer A. J. Ribeiro R. A. Hungria M. (2020). Genetic diversity of *Agrobacterium* species isolated from nodules of common bean and soybean in Brazil, Mexico, Ecuador and Mozambique, and description of the new species *Agrobacterium fabacearum* sp. nov. Int. J. Syst. Evol. Microbiol. 70, 4233–4244. 10.1099/ijsem.0.004278 32568030

[B23] Denton M. D. Phillips L. A. Peoples M. B. Pearce D. J. Swan A. D. Mele P. M.. (2017). Legume inoculant application methods: Effects on nodulation patterns, nitrogen fixation, crop growth and yield in narrow-leaf lupin and faba bean. Plant Soil 419, 25–39. 10.1007/s11104-017-3317-7

[B24] Dogra G. Purschke F. G. Wagner V. Haslbeck M. Kriehuber T. Hughes J. G.. (2012). *Sinorhizobium meliloti* CheA complexed with CheS exhibits enhanced binding to CheY1, resulting in accelerated CheY1 dephosphorylation. J. Bacteriol. 194, 1075–1087. 10.1128/JB.06505-11 22194454 PMC3294773

[B25] Ebrahimi A. N. Or D. (2014). Microbial dispersal in unsaturated porous media: Characteristics of motile bacterial cell motions in unsaturated angular pore networks. Water Resour. Res. 50, 7406–7429. 10.1002/2014wr015897

[B26] Eggenhofer E. Haslbeck M. Scharf B. (2004). MotE serves as a new chaperone specific for the periplasmic motility protein, MotC, in *Sinorhizobium meliloti*. Mol. Microbiol. 52, 701–712. 10.1111/j.1365-2958.2004.04022.x 15101977

[B27] Eggenhofer E. Rachel R. Haslbeck M. Scharf B. (2006). MotD of *Sinorhizobium meliloti* and related -proteobacteria is the flagellar-hook-length regulator and therefore reassigned as FliK. J. Bacteriol. 188, 2144–2153. 10.1128/JB.188.6.2144-2153.2006 16513744 PMC1428147

[B28] Felix G. Duran J. D. Volko S. Boller T. (1999). Plants have a sensitive perception system for the most conserved domain of bacterial flagellin. Plant J. 18, 265–276. 10.1046/j.1365-313X.1999.00265.x 10377992

[B29] Fujii M. Shibata S. Aizawa S.-I. (2008). Polar, peritrichous, and lateral flagella belong to three distinguishable flagellar families. J. Mol. Biol. 379, 273–283. 10.1016/j.jmb.2008.04.012 18455187

[B30] Galajda P. Keymer J. Chaikin P. Austin R. (2007). A wall of funnels concentrates swimming bacteria. J. Bacteriol. 189, 8704–8707. 10.1128/JB.01033-07 17890308 PMC2168927

[B31] Ganusova E. E. Vo L. T. Mukherjee T. Alexandre G. (2021). Multiple CheY proteins control surface-associated lifestyles of *Azospirillum brasilense*. Front. Microbiol. 12:664826. 10.3389/fmicb.2021.664826 33968002 PMC8100600

[B32] Gao S. Wu H. Yu X. Qian L. Gao X. (2016). Swarming motility plays the major role in migration during tomato root colonization by *Bacillus subtilis* SWR01. Biol. Control 98, 11–17. 10.1016/j.biocontrol.2016.03.011

[B33] Garrido-Sanz D. Redondo-Nieto M. Mongiardini E. Blanco-Romero E. Duron D. Quelas J. I.. (2019). Phylogenomic analyses of *Bradyrhizobium* reveal uneven distribution of the lateral and subpolar flagellar systems, which extends to rhizobiales. Microorganisms 7:50. 10.3390/microorganisms7020050 30781830 PMC6406911

[B34] Gotz R. Limmer N. Ober K. Schmitt R. (1982). Motility and chemotaxis in two strains of *Rhizobium* with complex flagella. Microbiology 128, 789–798. 10.1099/00221287-128-4-789

[B35] Griffin D. M. Quail G. (1968). Movement of bacteria in moist particulate systems. Aust. Jnl. Of Bio. Sci. 21, 579–582. 10.1071/bi9680579 5664141

[B36] Hamdi Y. A. (1971). Soil-water tension and the movement of rhizobia. Soil Biol. Biochem. 3, 121–126. 10.1016/0038-0717(71)90004-6

[B37] Henrichsen J. (1972). Bacterial surface translocation: a survey and a classification. Bacteriol. Rev. 36, 478–503. 10.1128/br.36.4.478-503.1972 4631369 PMC408329

[B38] Hirota N. Imae Y. (1983). Na+-driven flagellar motors of an alkalophilic *Bacillus* strain YN-1. J. Biol. Chem. 258, 10577–10581. 6885795

[B39] Ho S.-C. Wang J. L. Schindler M. Loh J. T. (1994). Carbohydrate binding activities of *Bradyrhizobium japonicum* III. Lectin expression, bacterial binding, and nodulation efficiency. Plant J. 5, 873–884. 10.1046/j.1365-313X.1994.5060873.x 8054992

[B40] Horiuchi J. Prithiviraj B. Bais H. P. Kimball B. A. Vivanco J. M. (2005). Soil nematodes mediate positive interactions between legume plants and rhizobium bacteria. Planta 222, 848–857. 10.1007/s00425-005-0025-y 16025342

[B41] Imam S. Chen Z. Roos D. S. Pohlschroder M. (2011). Identification of surprisingly diverse type IV pili, across a broad range of gram-positive bacteria. PLoS ONE 6:e28919. 10.1371/journal.pone.0028919 22216142 PMC3244431

[B42] Issa S. Wood M. Simmonds L. P. (1993). Active movement of chickpea and bean rhizobia in dry soil. Soil Biol. Biochem. 25, 951–958. 10.1016/0038-0717(93)90098-V

[B43] Iturralde E. T. Covelli J. M. Alvarez F. Perez-Gimenez J. Arrese-Igor C. Lodeiro A. R. (2019). Soybean-nodulating strains with low intrinsic competitiveness for nodulation, good symbiotic performance, and stress-tolerance isolated from soybean-cropped soils in Argentina. Front. Microbiol. 10:1061. 10.3389/fmicb.2019.01061 31139173 PMC6527597

[B44] Iwashkiw J. A. Vozza N. F. Kinsella R. L. Feldman M. F. (2013). Pour some sugar on it: the expanding world of bacterial protein O-linked glycosylation. Mol. Microbiol. 89, 14–28. 10.1111/mmi.12265 23679002

[B45] Jiang N. Liu W. Li Y. Xie Z. (2016). Comparative genomic and protein sequence analyses of the chemotaxis system of *Azorhizobium caulinodans*. Acta Microbiol. Sin. 56, 1256–1265. 10.13343/j.cnki.wsxb.20150500 29738195

[B46] Jimenez-Sanchez C. Wick L. Y. Cantos M. Ortega-Calvo J.-J. (2015). Impact of dissolved organic matter on bacterial tactic motility, attachment, and transport. Environ. Sci. Technol. 49, 4498–4505. 10.1021/es5056484 25734420

[B47] Jones C. W. Armitage J. P. (2015). Positioning of bacterial chemoreceptors. Trends Microbiol. 10.1016/j.tim.2015.03.004 25843366

[B48] Kanbe M. Yagasaki J. Zehner S. Gottfert M. Aizawa S.-I. (2007). Characterization of two sets of subpolar flagella in *Bradyrhizobium japonicum*. J. Bacteriol. 189, 1083–1089. 10.1128/JB.01405-06 17098908 PMC1797282

[B49] Kaneko T. Nakamura Y. Sato S. Minamisawa K. Uchiumi T. Sasamoto S.. (2002). Complete genomic sequence of nitrogen-fixing symbiotic bacterium *Bradyrhizobium japonicum* USDA110. DNA Res. 9, 189–197. 10.1093/dnares/9.6.189 12597275

[B50] Karmakar R. Chandra R. (2012). Effect of soil type and moisture content on survival, mobility, nodule occupancy of inoculated *Rhizobium leguminosarum* bv. *viciae* and lentil growth. Int. J. Agric. Environ. Biotechnol. 5, 7–12. Available online at: https://www.indianjournals.com/ijor.aspx?target=ijor:ijaeb&volume=5&issue=1&article=002

[B51] Karunakaran R. Ramachandran V. K. Seaman J. C. East A. K. Mouhsine B. Mauchline T. H.. (2009). Transcriptomic analysis of *Rhizobium leguminosarum* biovar *viciae* in symbiosis with host plants *Pisum sativum* and *Vicia cracca*. J. Bacteriol. 191, 4002–4014. 10.1128/JB.00165-09 19376875 PMC2698398

[B52] Kearns D. B. (2010). A field guide to bacterial swarming motility. Nat. Rev. Microbiol. 8, 634–644. 10.1038/nrmicro2405 20694026 PMC3135019

[B53] King W. L. Bell T. H. (2021). Can dispersal be leveraged to improve microbial inoculant success? Trends Biotechnol. 10.1016/j.tibtech.2021.04.008. [EPub ahead of print]. 33972105

[B54] Kojima S. Blair D. F. (2001). Conformational change in the stator of the bacterial flagellar motor. Biochemistry 40, 13041–13050. 10.1021/bi011263o 11669642

[B55] Krehenbrink M. Downie J. A. (2008). Identification of protein secretion systems and novel secreted proteins in *Rhizobium leguminosarum* bv. *viciae*. BMC Genomics 9:55. 10.1186/1471-2164-9-55 18230162 PMC2275737

[B56] Kristich C. J. Ordal G. W. (2002). *Bacillus subtilis* CheD is a chemoreceptor modification enzyme required for chemotaxis. J. Biol. Chem. 277, 25356–25362. 10.1074/jbc.M201334200 12011078

[B57] Lacal J. Garcia-Fontana C. Munoz-Martinez F. Ramos J. L. Krell T. (2010). Sensing of environmental signals: Classification of chemoreceptors according to the size of their ligand binding regions. Environ. Microbiol. 12, 2873–2884. 10.1111/j.1462-2920.2010.02325.x 20738376

[B58] Lauffenburger D. Aris R. Keller K. (1982). Effects of cell motility and chemotaxis on microbial population growth. Biophys. J. 40, 209–219. 10.1016/S0006-3495(82)84476-7 7183335 PMC1328997

[B59] Lee K.-B. De Backer P. Aono T. Liu C.-T. Suzuki S. Suzuki T.. (2008). The genome of the versatile nitrogen fixer *Azorhizobium caulinodans* ORS571. BMC Genomics 9:271. 10.1186/1471-2164-9-271 18522759 PMC2443382

[B60] Liu W. Bai X. Li Y. Min J. Kong Y. Hu X. (2020). CheY1 and CheY2 of *Azorhizobium caulinodans* ORS571 regulate chemotaxis and competitive colonization with the host plant. Appl. Environ. Microbiol. 86:e00599–20. 10.1128/AEM.00599-20 32471918 PMC7376556

[B61] Liu W. Sun Y. Shen R. Dang X. Liu X. Sui F.. (2018a). A chemotaxis-like pathway of *Azorhizobium caulinodans* controls flagella-driven motility, which regulates biofilm formation, exopolysaccharide biosynthesis, and competitive nodulation. Mol. Plant. Microbe Interact. 31, 737–749. 10.1094/MPMI-12-17-0290-R 29424664

[B62] Liu X. Liu W. Sun Y. Xia C. Elmerich C. Xie Z. (2018b). A *cheZ*-like gene in *Azorhizobium caulinodans* is a key gene in the control of chemotaxis and colonization of the host plant. Appl. Environ. Microbiol. 84:e01827–17. 10.1128/AEM.01827-17 29150498 PMC5772239

[B63] Liu X. Xie Z. Wang Y. Sun Y. Dang X. Sun H. (2019). A dual role of amino acids from *Sesbania rostrata* seed exudates in the chemotaxis response of *Azorhizobium caulinodans* ORS571. Mol. Plant. Microbe Interact. 32, 1134–1147. 10.1094/MPMI-03-19-0059-R 30920344

[B64] Loh J. T. Ho S. C. de Feijter A. W. Wang J. L. Schindler M. (1993). Carbohydrate binding activities of *Bradyrhizobium japonicum*: unipolar localization of the lectin BJ38 on the bacterial cell surface. Proc. Natl. Acad. Sci. U.S.A. 90, 3033–3037. 10.1073/pnas.90.7.3033 8464919 PMC46231

[B65] Lopez-Farfan D. Reyes-Darias J. A. Matilla M. A. Krell T. (2019). Concentration dependent effect of plant root exudates on the chemosensory systems of *Pseudomonas putida* KT2440. Front. Microbiol. 10:78. 10.3389/fmicb.2019.00078 30761113 PMC6363813

[B66] Lopez-Garcia S. L. Perticari A. Piccinetti C. Ventimiglia L. Arias N. Battista J. J. D.. (2009). In-furrow inoculation and selection for higher motility enhances the efficacy of *Bradyrhizobium japonicum* nodulation. Agron. J. 101, 357–363. 10.2134/agronj2008.0155x

[B67] Lopez-Garcia S. L. Vezquez T. E. E. Favelukes G. Lodeir A. R. (2002). Rhizobial position as a main determinant in the problem of competition for nodulation in soybean. Environ. Microbiol. 4, 216–224. 10.1046/j.1462-2920.2002.00287.x 12010128

[B68] Ma L. Shi Y. Siemianowski O. Yuan B. Egner T. K. Mirnezami S. V.. (2019). Hydrogel-based transparent soils for root phenotyping *in vivo*. Proc. Natl. Acad. Sci. U.S.A. 116, 11063–11068. 10.1073/pnas.1820334116 31088969 PMC6561166

[B69] Macnab R. M. Aizawa S. I. (1984). Bacterial motility and the bacterial flagellar motor. Annu. Rev. Biophys. Bioeng. 13, 51–83. 10.1146/annurev.bb.13.060184.000411. 6378075

[B70] Madsen E. L. Alexander M. (1982). Transport of rhizobium and pseudomonas through soil. Soil Sci. Soc. Am. J. 46, 557–560. 10.2136/sssaj1982.03615995004600030023x

[B71] Mahler R. L. Wollum A. G. (1981). The influence of soil water potential and soil texture on the survival of *Rhizobium japonicum* and *Rhizobium leguminosarum* isolates in the soil. Soil Sci. Soc. Am. J. 45, 761–766. 10.2136/sssaj1981.03615995004500040017x

[B72] Mao H. Cremer P. S. Manson M. D. (2003). A sensitive, versatile microfluidic assay for bacterial chemotaxis. Proc. Natl. Acad. Sci. U.S.A. 100, 5449–5454. 10.1073/pnas.0931258100 12704234 PMC154365

[B73] Mattick J. S. (2002). Type IV pili and twitching motility. Annu. Rev. Microbiol. 56, 289–314. 10.1146/annurev.micro.56.012302.160938 12142488

[B74] McCarter L. L. (2006). Regulation of flagella. Curr. Opin. Microbiol. 9, 180–186. 10.1016/j.mib.2006.02.001 16487743

[B75] McDermott T. R. Graham P. H. (1989). *Bradyrhizobium japonicum* inoculant mobility, nodule occupancy, and acetylene reduction in the soybean root system. Appl. Environ. Microbiol. 55, 2493–2498. 16348026 10.1128/aem.55.10.2493-2498.1989 PMC203110

[B76] Meade H. M. Long S. R. Ruvkun G. B. Brown S. E. Ausubel F. M. (1982). Physical and genetic characterization of symbiotic and auxotrophic mutants of *Rhizobium meliloti* induced by transposon Tn5 mutagenesis. J. Bacteriol. 149, 114–122. 10.1128/jb.149.1.114-122.1982 6274841 PMC216598

[B77] Meier V. M. Muschler P. Scharf B. E. (2007). Functional analysis of nine putative chemoreceptor proteins in *Sinorhizobium meliloti*. J. Bacteriol. 189, 1816–1826. 10.1128/JB.00883-06 17189365 PMC1855726

[B78] Meier V. M. Scharf B. E. (2009). Cellular localization of predicted transmembrane and soluble chemoreceptors in *Sinorhizobium meliloti*. J. Bacteriol. 191, 5724–5733. 10.1128/JB.01286-08 19617359 PMC2737976

[B79] Mendoza-Suarez M. A. Geddes B. A. Sanchez-Canizares C. Ramarez-Gonzalez R. H. Kirchhelle C. Jorrin B. Poole P. S. (2020). Optimizing Rhizobium-legume symbioses by simultaneous measurement of rhizobial competitiveness and N2 fixation in nodules. Proc. Natl. Acad. Sci. U.S.A. 117, 9822–9831. 10.1073/pnas.1921225117 32317381 PMC7211974

[B80] Miller L. D. Yost C. K. Hynes M. F. Alexandre G. (2007). The major chemotaxis gene cluster of *Rhizobium leguminosarum* bv. *viciae* is essential for competitive nodulation. Mol. Microbiol. 63, 348–362. 10.1111/j.1365-2958.2006.05515.x 17163982

[B81] Mitchell J. G. Pearson L. Dillon S. (1996). Clustering of marine bacteria in seawater enrichments. Appl. Environ. Microbiol. 62, 3716–3721. 10.1128/aem.62.10.3716-3721.1996 16535420 PMC1388958

[B82] Moens S. Michiels K. Keijers V. Van Leuven F. Vanderleyden J. (1995a). Cloning, sequencing, and phenotypic analysis of Laf1, encoding the flagellin of the lateral flagella of *Azospirillum brasilense* Sp7. J. Bacteriol. 177, 5419–5426. 7559324 10.1128/jb.177.19.5419-5426.1995 PMC177346

[B83] Moens S. Michiels K. Vanderleyden J. (1995b). Glycosylation of the flagellin of the polar flagellum of *Azospirillum brasilense*, a Gram-negative nitrogen-fixing bacterium. Microbiology 141, 2651–2657. 10.1099/13500872-141-10-2651

[B84] Mongiardini E. J. Parisi G. D. Quelas J. I. Lodeiro A. R. (2016). The tight-adhesion proteins TadGEF of *Bradyrhizobium diazoefficiens* USDA 110 are involved in cell adhesion and infectivity on soybean roots. Microbiol. Res. 182, 80–88. 10.1016/j.micres.2015.10.001 26686616

[B85] Mongiardini E. J. Quelas J. I. Dardis C. Althabegoiti M. J. Lodeiro A. R. (2017). Transcriptional control of the lateral-flagellar genes of *Bradyrhizobium diazoefficiens*. J. Bacteriol. 199, e00253–17. 10.1128/JB.00253-17 28533217 PMC5512216

[B86] Mukherjee T. Elmas M. Vo L. Alexiades V. Hong T. Alexandre G. (2019). Multiple CheY homologs control swimming reversals and transient pauses in *Azospirillum brasilense*. Biophys. J. 116, 1527–1537. 10.1016/j.bpj.2019.03.006 30975454 PMC6486476

[B87] Mukherjee T. Kumar D. Burriss N. Xie Z. Alexandre G. (2016). *Azospirillum brasilense* chemotaxis depends on two signaling pathways regulating distinct motility parameters. J. Bacteriol. 198, 1764–1772. 10.1128/JB.00020-16 27068592 PMC4886762

[B88] Nan B. Zusman D. R. (2016). Novel mechanisms power bacterial gliding motility. Mol. Microbiol. 101, 186–193. 10.1111/mmi.13389 27028358 PMC5008027

[B89] Nedeljkovia M. Sastre D. E. Sundbe E. J. (2021). Bacterial flagellar filament: a supramolecular multifunctional nanostructure. Int. J. Mol. Sci. 22:7521. 10.3390/ijms22147521 34299141 PMC8306008

[B90] Parke J. L. Moen R. Rovira A. D. Bowen G. D. (1986). Soil water flow affects the rhizosphere distribution of a seed-borne biological control agent, *Pseudomonas fluorescens*. Soil Biol. Biochem. 18, 583–588. 10.1016/0038-0717(86)90079-9

[B91] Pollitt E. J. G. Diggle S. P. (2017). Defining motility in the *Staphylococci*. Cell. Mol. Life Sci. 74, 2943–2958. 10.1007/s00018-017-2507-z 28378043 PMC5501909

[B92] Porter S. L. Wadhams G. H. Armitage J. P. (2011). Signal processing in complex chemotaxis pathways. Nat. Rev. Microbiol. 9, 153–165. 10.1038/nrmicro2505 21283116

[B93] Quelas J. I. Althabegoiti M. J. Jimenez-Sanchez C. Melgarejo A. A. Marconi V. I. Mongiardini E. J.. (2016). Swimming performance of *Bradyrhizobium diazoefficiens* is an emergent property of its two flagellar systems. Sci. Rep. 6:23841. 10.1038/srep23841 27053439 PMC4823718

[B94] Raina J.-B. Fernandez V. Lambert B. Stocker R. Seymour J. R. (2019). The role of microbial motility and chemotaxis in symbiosis. Nat. Rev. Microbiol. 17, 284–294. 10.1038/s41579-019-0182-9 30923350

[B95] Rao C. V. Glekas G. D. Ordal G. W. (2008). The three adaptation systems of *Bacillus subtilis* chemotaxis. Trends Microbiol. 16, 480–487. 10.1016/j.tim.2008.07.003 18774298 PMC3532902

[B96] Rebbapragada A. Johnson M. S. Harding G. P. Zuccarelli A. J. Fletcher H. M. Zhulin I. B.. (1997). The Aer protein and the serine chemoreceptor Tsr independently sense intracellular energy levels and transduce oxygen, redox, and energy signals for *Escherichia coli* behavior. Proc. Natl. Acad. Sci. U.S.A. 94, 10541–10546. 10.1073/pnas.94.20.10541 9380671 PMC23396

[B97] Rice M. S. Dahlquist F. W. (1991). Sites of deamidation and methylation in Tsr, a bacterial chemotaxis sensory transducer. J. Biol. Chem. 266, 9746–9753. 10.1007/s00249-007-0247-y 2033064

[B98] Rossi F. A. Medeot D. B. Liaudat J. P. Pistorio M. Jofre E. (2016). In *Azospirillum brasilense*, mutations in *flmA* or *flmB* genes affect polar flagellum assembly, surface polysaccharides, and attachment to maize roots. Microbiol. Res. 190, 55–62. 10.1016/j.micres.2016.05.006 27393999

[B99] Rotter C. Muhlbacher S. Salamon D. Schmitt R. Scharf B. (2006). Rem, a new transcriptional activator of motility and chemotaxis in *Sinorhizobium meliloti*. J. Bacteriol. 188, 6932–6942. 10.1128/JB.01902-05 16980496 PMC1595514

[B100] Scharf B. Schuster-Wolff-Bhring H. Rachel R. Schmitt R. (2001). Mutational analysis of the *Rhizobium lupini* H13-3 and *Sinorhizobium meliloti* flagellin genes: Importance of flagellin A for flagellar filament structure and transcriptional regulation. J. Bacteriol. 183, 5334–5342. 10.1128/JB.183.18.5334-5342.2001 11514517 PMC95416

[B101] Scharf B. E. Hynes M. F. Alexandre G. M. (2016). Chemotaxis signaling systems in model beneficial plant-bacteria associations. Plant Mol. Biol. 90, 549–559. 10.1007/s11103-016-0432-4 26797793

[B102] Sepehrnia N. Bachmann J. Hajabbasi M. A. Rezanezhad F. Lichner L. Hallett P. D.. (2019). Transport, retention, and release of *Escherichia coli* and *Rhodococcus erythropolis* through dry natural soils as affected by water repellency. Sci. Total Environ. 694:133666. 10.1016/j.scitotenv.2019.133666 31394325

[B103] Shelud'ko A. V. Katsy E. I. (2001). Formation of polar bundles of pili and the behavior of *Azospirillum brasilense* cells in a semiliquid Agar. Microbiology 70, 570–575. 10.1023/A:1012364323315 11763787

[B104] Simons M. van der Bij A. J. Brand I. de Weger L. A. Wijffelman C. A. Lugtenberg B. J. J. (1996). Gnotobiotic system for studying rhizosphere colonization by plant growth-promoting *Pseudomonas* bacteria. Mol. Plant Microbe Interact. 9, 600–607. 10.1094/mpmi-9-0600 8810075

[B105] Singh T. Srivastava A. K. Arora D. K. (2002). Horizontal and vertical movement of *Pseudomonas fluorescens* toward exudate of *Macrophomina phaseolina* in soil: influence of motility and soil properties. Microbiol. Res. 157, 139–148. 10.1078/0944-5013-00142 12002402

[B106] Son K. Guasto J. S. Stocker R. (2013). Bacteria can exploit a flagellar buckling instability to change direction. Nat. Phys. 9, 494–498. 10.1038/nphys2676

[B107] Sourjik V. Muschler P. Scharf B. Schmitt R. (2000). VisN and VisR are global regulators of chemotaxis, flagellar, and motility genes in *Sinorhizobium* (*Rhizobium*) *meliloti*. J. Bacteriol. 182, 782–788. 10.1128/JB.182.3.782-788.2000 10633114 PMC94343

[B108] Sourjik V. Schmitt R. (1998). Phosphotransfer between CheA, CheY1, and CheY2 in the chemotaxis signal transduction chain of *Rhizobium meliloti*. Biochemistry 37, 2327–2335. 10.1021/bi972330a 9485379

[B109] Sourjik V. Wingreen N. S. (2012). Responding to chemical gradients: Bacterial chemotaxis. Curr. Opin. Cell Biol. 24, 262–268. 10.1016/j.ceb.2011.11.008 22169400 PMC3320702

[B110] Stock D. Namba K. Lee L. K. (2012). Nanorotors and self-assembling macromolecular machines: The torque ring of the bacterial flagellar motor. Curr. Opin. Biotechnol. 23, 545–554. 10.1016/j.copbio.2012.01.008 22321941

[B111] Taguchi F. Suzuki T. Takeuchi K. Inagaki Y. Toyoda K. Shiraishi T. Ichinose Y. (2009). Glycosylation of flagellin from *Pseudomonas syringae* pv. *tabaci* 6605 contributes to evasion of host tobacco plant surveillance system. Physiol. Mol. Plant Pathol. 74, 11–17. 10.1016/j.pmpp.2009.08.001

[B112] Takeuchi K. Taguchi F. Inagaki Y. Toyoda K. Shiraishi T. Ichinose Y. (2003). Flagellin glycosylation island in *Pseudomonas syringae* pv. *glycinea* and its role in host specificity. J. Bacteriol. 185, 6658–6665. 10.1128/JB.185.22.6658-6665.2003 14594840 PMC262107

[B113] Taktikos J. Stark H. Zaburdaev V. (2013). How the Motility Pattern of Bacteria Affects Their Dispersal and Chemotaxis. PLoS ONE 8:e81936. 10.1371/journal.pone.0081936 24391710 PMC3876982

[B114] Tambalo D. D. Bustard D. E. Del Bel K. L. Koval S. F. Khan M. F. Hynes M. F. (2010a). Characterization and functional analysis of seven flagellin genes in *Rhizobium leguminosarum* bv. *viciae*. BMC Microbiol. 10:219. 10.1186/1471-2180-10-219 20716375 PMC2936354

[B115] Tambalo D. D. Del Bel K. L. Bustard D. E. Greenwood P. R. Steedman A. E. Hynes M. F. (2010b). Regulation of flagellar, motility and chemotaxis genes in *Rhizobium leguminosarum* by the VisN/R-Rem cascade. Microbiology 156, 1673–1685. 10.1099/mic.0.035386-0 20203055

[B116] Tecon R. Or D. (2017). Biophysical processes supporting the diversity of microbial life in soil. FEMS Microbiol. Rev. 41, 599–623. 10.1093/femsre/fux039 28961933 PMC5812502

[B117] Terahara N. Sano M. Ito M. (2012). A *Bacillus* flagellar motor that can use both Na+ and K+ as a coupling ion is converted by a single mutation to use only Na+. PLoS ONE 7:e46248. 10.1371/journal.pone.0046248 23049994 PMC3457975

[B118] Tomich M. Planet P. J. Figurski D. H. (2007). The *Tad* locus: postcards from the widespread colonization island. Nat. Rev. Microbiol. 5, 363–375. 10.1038/nrmicro1636 17435791

[B119] Turnbull G. A. Morgan J. A. W. Whipps J. M. Saunders J. R. (2001). The role of bacterial motility in the survival and spread of *Pseudomonas fluorescens* in soil and in the attachment and colonisation of wheat roots. FEMS Microbiol. Ecol. 36, 21–31. 10.1111/j.1574-6941.2001.tb00822.x 11377770

[B120] van Elsas J. D. Trevors J. T. van Overbeek L. S. (1991). Influence of soil properties on the vertical movement of genetically-marked *Pseudomonas fluorescens* through large soil microcosms. Biol. Fert. Soils 10, 249–255. 10.1007/BF00337375

[B121] Wadisirisuk P. Danso S. K. A. Hardarson G. Bowen G. D. (1989). Influence of *Bradyrhizobium japonicum* location and movement on nodulation and nitrogen fixation in soybeans. Appl. Environ. Microbiol. 55, 1711–1716. 16347964 10.1128/aem.55.7.1711-1716.1989 PMC202939

[B122] Walsh E. J. Feuerborn A. Wheeler J. H. R. Tan A. N. Durham W. M. Foster K. R. Cook P. R. (2017). Microfluidics with fluid walls. Nat. Commun. 8:816. 10.1038/s41467-017-00846-4 29018186 PMC5635017

[B123] Wang W. Jiang Z. Westermann M. Ping L. (2012). Three mutations in *Escherichia coli* that generate transformable functional flagella. J. Bacteriol. 194, 5856–5863. 10.1128/JB.01102-12 22923592 PMC3486126

[B124] Webb B. A. Compton K. K. del Campo J. S. M. Taylor D. Sobrado P. Scharf B. E. (2017a). *Sinorhizobium meliloti* chemotaxis to multiple amino acids is mediated by the chemoreceptor McpU. Mol. Plant Microbe Interact. 30, 770–777. 10.1094/MPMI-04-17-0096-R 28745538

[B125] Webb B. A. Helm R. F. Scharf B. E. (2016). Contribution of individual chemoreceptors to *Sinorhizobium meliloti* chemotaxis towards amino acids of host and nonhost seed exudates. Mol. Plant Microbe Interact. 29, 231–239. 10.1094/MPMI-12-15-0264-R 26713349

[B126] Webb B. A. Karl Compton K. Castaneda Saldana R. Arapov T. D. Keith Ray W. Helm R. F.. (2017b). *Sinorhizobium meliloti* chemotaxis to quaternary ammonium compounds is mediated by the chemoreceptor McpX. Mol. Microbiol. 103, 333–346. 10.1111/mmi.13561 27748981

[B127] Wheatley R. M. Ford B. L. Li L. Aroney S. T. N. Knights H. E. Ledermann R.. (2020). Lifestyle adaptations of *Rhizobium* from rhizosphere to symbiosis. Proc. Natl. Acad. Sci. U.S.A. 117, 23823–23834. 10.1073/pnas.2009094117 32900931 PMC7519234

[B128] Wibberg D. Blom J. Jaenicke S. Kollin F. Rupp O. Scharf B.. (2011). Complete genome sequencing of *Agrobacterium* sp. H13-3, the former *Rhizobium lupini* H13-3, reveals a tripartite genome consisting of a circular and a linear chromosome and an accessory plasmid but lacking a tumor-inducing Ti-plasmid. J. Biotechnol. 155, 50–62. 10.1016/j.jbiotec.2011.01.010 21329740

[B129] Wisniewski-Dye F. Borziak K. Khalsa-Moyers G. Alexandre G. Sukharnikov L. O. Wuichet K.. (2011). *Azospirillum* genomes reveal transition of bacteria from aquatic to terrestrial environments. PLoS Genet. 7:e1002430. 10.1371/journal.pgen.1002430 22216014 PMC3245306

[B130] Worrall V. Roughley R. J. (1991). Vertical movement of *Rhizobium leguminosarum* bv. *trifolii* in soil as influenced by soil water potential and water flow. Soil Biol. Biochem. 23, 485–486. 10.1016/0038-0717(91)90014-B

[B131] Worrall V. S. Roughley R. J. (1976). The effect of moisture stress on infection of *Trifolium subterraneum* L. By *Rhizobium trifolii* Dang. J. Exp. Bot. 27, 1233–1241. 10.1093/jxb/27.6.1233

[B132] Wuichet K. Zhulin I. B. (2010). Origins and diversification of a complex signal transduction system in prokaryotes. Sci. Signal. 3, 1–14. 10.1126/scisignal.2000724 20587806 PMC3401578

[B133] Xie L. Altindal T. Chattopadhyay S. Wu X.-L. (2011). Bacterial flagellum as a propeller and as a rudder for efficient chemotaxis. Proc. Natl. Acad. Sci. U.S.A. 108, 2246–2251. 10.1073/pnas.1011953108 21205908 PMC3038696

[B134] Yang P. van Elsas J. D. (2018). Mechanisms and ecological implications of the movement of bacteria in soil. Appl. Soil Ecol. 129, 112–120. 10.1016/j.apsoil.2018.04.014

[B135] Yost C. K. (1998). Characterization of *Rhizobium leguminosarum* genes homologous to chemotaxis chemoreceptors.

[B136] Yost C. K. Del Bel K. L. Quandt J. Hynes M. F. (2004). *Rhizobium leguminosarum* methyl-accepting chemotaxis protein genes are down-regulated in the pea nodule. Arch. Microbiol. 182, 505–513. 10.1007/s00203-004-0736-7 15502966

[B137] Yost C. K. Rochepeau P. Hynes M. F. (1998). *Rhizobium leguminosarum* contains a group of genes that appear to code for methyl-accepting chemotaxis proteins. Microbiology 144, 1945–1956. 10.1099/00221287-144-7-1945 9695927

[B138] Young J. P. W. Crossman L. C. Johnston A. W. B. Thomson N. R. Ghazoui Z. F. Hull K. H.. (2006). The genome of *Rhizobium leguminosarum* has recognizable core and accessory components. Genome Biol. 7. 10.1186/gb-2006-7-4-r34 16640791 PMC1557990

[B139] Zatakia H. M. Arapov T. D. Meier V. M. Scharf B. E. (2017). Cellular stoichiometry of methyl-accepting chemotaxis proteins inx *Sinorhizobium meliloti*. J. Bacteriol. 200:e00614–17. 10.1128/JB.00614-17 29263102 PMC5826028

[B140] Zatakia H. M. Nelson C. E. Syed U. J. Scharf B. E. (2014). ExpR coordinates the expression of symbiotically important, bundle-forming Flp pili with quorum sensing in *Sinorhizobium meliloti*. Appl. Environ. Microbiol. 80, 2429–2439. 10.1128/AEM.04088-13 24509921 PMC3993167

[B141] Zhuang X.-Y. Lo C.-J. (2020). Construction and loss of bacterial flagellar filaments. Biomolecules 10:1528. 10.3390/biom10111528 33182435 PMC7696725

[B142] Zhulin I. B. Armitage J. P. (1993). Motility, chemokinesis, and methylation-independent chemotaxis in *Azospirillum brasilense*. J. Bacteriol. 175, 952–958. 8432718 10.1128/jb.175.4.952-958.1993 PMC193006

